# When Non-Dominant Is Better than Dominant: Kinesiotape Modulates Asymmetries in Timed Performance during a Synchronization-Continuation Task

**DOI:** 10.3389/fnint.2017.00021

**Published:** 2017-09-08

**Authors:** Riccardo Bravi, Erez J. Cohen, Alessio Martinelli, Anna Gottard, Diego Minciacchi

**Affiliations:** ^1^Department of Experimental and Clinical Medicine, University of Florence Florence, Italy; ^2^Department of Information Engineering, University of Florence Florence, Italy; ^3^Department of Statistics, Informatics, Applications, University of Florence Florence, Italy

**Keywords:** sensorimotor integration, timing of action, augmented feedback, human motor control, laterality of motor control

## Abstract

There is a growing consensus regarding the specialization of the non-dominant limb (NDL)/hemisphere system to employ proprioceptive feedback when executing motor actions. In a wide variety of rhythmic tasks the dominant limb (DL) has advantages in speed and timing consistency over the NDL. Recently, we demonstrated that the application of Kinesio^®^ Tex (KT) tape, an elastic therapeutic device used for treating athletic injuries, improves significantly the timing consistency of isochronous wrist’s flexion-extensions (IWFEs) of the DL. We argued that the augmented precision of IWFEs is determined by a more efficient motor control during movements due to the extra-proprioceptive effect provided by KT. In this study, we tested the effect of KT on timing precision of IWFEs performed with the DL and the NDL, and we evaluated the efficacy of KT to counteract possible timing precision difference between limbs. Young healthy subjects performed with and without KT (NKT) a synchronization-continuation task in which they first entrained IWFEs to paced auditory stimuli (synchronization phase), and subsequently continued to produce motor responses with the same temporal interval in the absence of the auditory stimulus (continuation phase). Two inter-onset intervals (IOIs) of 550-ms and 800-ms, one within and the other beyond the boundaries of the spontaneous motor tempo, were tested. Kinematics was recorded and temporal parameters were extracted and analyzed. Our results show that limb advantages in performing proficiently rhythmic movements are not side-locked but depend also on speed of movement. The application of KT significantly reduces the timing variability of IWFEs performed at 550-ms IOI. KT not only cancels the disadvantages of the NDL but also makes it even more precise than the DL without KT. The superior sensitivity of the NDL to use the extra-sensory information provided by KT is attributed to a greater competence of the NDL/hemisphere system to rely on sensory input. The findings in this study add a new piece of information to the context of motor timing literature. The performance asymmetries here demonstrated as preferred temporal environments could reflect limb differences in the choice of sensorimotor control strategies for the production of human movement.

## Introduction

Motor timing coordination is referred to the ability of individuals to perceive and generate motor responses at appropriate time intervals (Buhusi and Meck, 2005), and, like any motor behavior, it is characterized by some degree of variability (Fitts, 1954). The extent of this variability varies depending on the type of task performed, but also on the limb performing the task. In fact, behavioral research has revealed numerous advantages of the dominant (or preferred) limb in the generation of motor output including an increased strength (Armstrong and Oldham, 1999; Farthing et al., 2005), rate (Todor and Kyprie, 1980; Noguchi et al., 2006) and consistency of movement (Peters, 1976; Todor et al., 1982; Schmidt et al., 2000). For instance, Armstrong and Oldham (1999), when comparing maximum grip forces in healthy subjects, showed that they produced with the dominant arm forces approximately larger by 10% than those produced with the non-dominant arm. Also, in reaching tasks, when the rate by which pegs can be moved on a pegboard was evaluated, the dominant hand showed to be superior to the non-dominant one (Noguchi et al., 2006). Moreover, numerous finger tapping experiments have demonstrated that the dominant limb (DL) has advantages, in terms of speed and timing consistency, over the non-dominant limb (NDL) when sequential actions are performed at maximal speed (Peters, 1976; Todor et al., 1982; Schmidt et al., 2000). This asymmetric motor skill in favor of DL has been explained not only by increased use and training of the hand muscles (Ozcan et al., 2004) but also by the relatively enlarged excitability of the dominant motor cortex (De Gennaro et al., 2004) as well as by the increased excitability of motor-neuronal pool at the level of spinal circuitry (Adam et al., 1998).

The execution of a motor task may also be influenced by the different senses of the somatosensory system (Avanzino and Fiorio, 2014). Proprioception, defined as the ability to sense the position and the movement of a limb in space along with muscular effort and tension (Proske and Gandevia, 2009), is surely central to determine the accuracy of motor performance (Guigon et al., 2008; Rosenkranz et al., 2009). Specifically to timing control, evidence for the relevance of somatosensory feedback in timing coordination comes from studies that investigated basic mechanisms of timing by using a tapping paradigm. In fact, damage to peripheral or central structures for sensory/proprioceptive information processing results in the increase of timing variability. For instance, timing skills were found impaired in a deafferented patient respect to those of healthy subjects (LaRue et al., 1995). Also, Spencer et al. (2003) showed a deleterious reduction of timing precision in individuals with cerebellar lesions, a nervous structure that is strongly implicated in the processing of proprioceptive information (Tinazzi et al., 2013).

Given that sensory feedback plays an essential role in motor control, it is logical to hypothesize that the use of some device, able to influence proprioceptive information, may result in a modification of the performance precision. We examined the effect of the Kinesio^®^ Tex (KT) tape, as a sensory device, on influencing the precision of motor timing coordination. KT taping is a kinesthetic method currently used in clinical practice to benefit some symptoms of athletic injuries and a variety of physical disorders (Kase et al., 2013). Developed by Japanese chiropractor Dr. Kenso Kase in the 1970’s (Morris et al., 2013), KT is a specially designed tape having elastic properties and stretching capability with the purpose of mimicking the thickness and flexibility of the skin (Kase et al., 2013). It is claimed that KT application provides, while the movement occurs, a constant pulling force to the skin over which it is applied and brings about a lifting of the skin away from the tissue beneath, favoring the release of pressure from tender tissues underneath (Morris et al., 2013). Recently, a magnetic resonance imaging study quantified objectively KT mechanical effects on the skin and soft tissues over which it is applied (Pamuk and Yucesoy, 2015).

KT application was shown to influence significantly proprioception (Pelosin et al., 2013; Seo et al., 2016). KT was found to induce a modification in the ability of sensory discrimination, which is abnormal in patients with dystonia (Pelosin et al., 2013). Moreover, Seo et al. (2016) found that, in normal adults with sprained ankles, KT improved position sense in the dorsiflexion and inversion of the ankle joint. At first it was proposed that extra-proprioceptive effect provided by KT is due to the stimulation of cutaneous mechanoreceptors via stretching/deformation of skin (Kase et al., 2013). However, the recent study by Pamuk and Yucesoy (2015) showed that KT application causes deformations of targeted and deeper muscle tissues and permitted to make more plausible the assumption that KT may also stimulate muscle spindles during movement (Chang et al., 2010).

Recently, our group has devoted attention to improve the understanding of how KT is able to modulate motor control, and namely the variability, of a rhythmic motor behavior. We investigated the effect of KT application on timing coordination in healthy individuals by studying repeated isochronous wrist’s flexion-extensions (IWFEs) performed with no direct surface opposition and while minimizing visual information (Bravi et al., 2014a,b, 2015, 2016). We showed that KT, when applied on the dominant arm, was able to reduce timing variability of IWFEs performed under various auditory conditions and during their recall (Bravi et al., 2014b). In addition, we showed that sensorimotor coordination was significantly improved independently from direction and tension of KT application (Bravi et al., 2016). We attributed the effect of KT to augmented afferent proprioceptive information via the stimulation of mechanoreceptors.

Notwithstanding the dominant upper right limb was found to be faster (Flowers, 1975; Elliott et al., 1999), more accurate (Carson et al., 1993) and less variable (Elliott et al., 1999) than the non-dominant left arm, there is a growing consensus regarding the specialization of the NDL/hemisphere system for utilizing proprioceptive feedback (Colley, 1984; Riolo-Quinn, 1991; Goble et al., 2006; Goble and Brown, 2007, 2010). Conversely, the dominant system was suggested to more likely function in a feedforward fashion (Goble and Brown, 2007). While over the past decades between-hand differences during rhythmic cyclical movements have been explored quite in depth (Peters, 1976; Todor et al., 1982; Schmidt et al., 2000), how flexible they are, and whether and by what means these differences can be modulated is much less known. Therefore, in the current study we aimed to investigate the impact of KT when applied on DL and NDL on timing variability of IWFEs.

Furthermore, spontaneous rhythmic activity is a pervasive behavior of the nervous system in animals and humans (Brown, 1914; Yates et al., 1972; Sternad et al., 2000). Spontaneous motor tempo is defined as the frequency that a moving organism prefers when performing rhythmic actions (MacDougall and Moore, 2005). Although each individual has its own spontaneous tempo, it was shown that humans prefer to perform rhythmic motor behaviors with different motor effectors within a frequency region around 2 Hz, ranging from 2.2 Hz to 1.66 Hz (Vanneste et al., 2001; MacDougall and Moore, 2005; McAuley et al., 2006; Bisio et al., 2015). Spontaneous motor tempo is speculated to reflect the intrinsic rate of a spinal central generator (MacDougall and Moore, 2005). Central patterns generators (CPGs) are spinal neuronal networks that are thought to contribute to the execution of rhythmic motor patterns, such as locomotion, by generation of periodic motor commands (Frigon et al., 2004; Zehr et al., 2007). While CPGs have been well ascertained in invertebrates, primitive fish, and quadrupeds like cats (Arshavsky et al., 1985; Grillner, 1985; Baev et al., 1991), it is hard to locate elements of such circuits in higher vertebrates due to the complexity of the nervous structures and their additional modulation by higher brain centers (Schaal et al., 2004). Although the existence of CPGs in humans is only inferred indirectly, recent evidences suggest that neuronal networks are generally well preserved throughout evolution (Lamb and Yang, 2000; Marder, 2001; Zehr et al., 2007; Guertin, 2013).

We previously found that the reduction of timing variability of IWFEs provided by KT is concomitant with the modulation of neural processes elicited to govern the temporal production of rhythmic movements (Bravi et al., 2014b). Specifically, mean lag-1 autocorrelation values were biased towards positive when KT was applied, indicating a reinforcement of dynamic control of non-temporal movement parameters (Spencer and Ivry, 2005; Huys et al., 2008). This allows us to suspect that the application of KT, by augmenting proprioceptive information during movement, reinforces the efficiency of spinal motor circuitry, rendering the production of IWFEs less dependent on central drive (Bravi et al., 2014b). Therefore, to pursue our hypothesis, we evaluated the effect of KT on sets of IWFEs having interval duration of 550-ms and 800-ms (equivalent in that order to 1.81 Hz and 1.25 Hz). These durations were chosen since we were interested to investigate two movement frequencies falling, respectively, within and beyond the boundaries of the spontaneous motor tempo.

In this study, our interest was the assessment of an inexpensive wearable sensory device like KT in influencing rhythmic motor behavior. Specifically, we tested the effect of KT on timing variability of IWFEs performed with the DL and the NDL, and we evaluated the efficacy of KT to counteract possible timing precision difference between limbs. Also, since past numerous experiments have evidenced the superiority of DL over the NDL when sequential actions are performed at maximal speed (Peters, 1976; Todor et al., 1982; Schmidt et al., 2000), this study gives the opportunity to test whether such timing precision asymmetry is still preserved when the speed of rhythmic movement is not maximal.

We thus performed an experiment in which healthy subjects, tested with KT and without KT (NKT), have participated in two sessions (KT and NKT cases) in which sets of IWFEs were performed with the DL and the NDL, in a synchronization-continuation task at the two inter-onset intervals (IOIs) of 550-ms and 800-ms. As in our previous studies (Bravi et al., 2014a,b, 2015, 2016) participants were asked to perform movements in a natural way (Huys et al., 2008).

Our first experimental hypothesis is that the effect of KT should be greater on NDL since NDL/hemisphere system is specialized for utilizing proprioceptive feedback (Goble et al., 2006; Goble and Brown, 2007, 2010). Additionally, in the event of a specific action of KT on spinal circuitry, we expect to observe more prominent effect of KT when participants perform movements within the spontaneous motor tempo range of frequency (MacDougall and Moore, 2005; McAuley et al., 2006).

## Materials and Methods

### Participants

Twenty-five healthy adults were recruited for this study (age: 22.7 ± 2.5 years; 12 males and 13 females). All participants were right handed (82.1 ± 23.4; laterality score from the Edinburgh Handedness Inventory, Oldfield, 1971); they were naive to the task and the purpose of the study, and knew nothing about the KT method. All were not musically trained and free of documented auditory, motor, neurological impairments. Participants were not paid. The study protocol was approved by the Institutional Ethics Committee (Comitato Etico Area Vasta Centro AOUCareggi, Florence, Italy; Prot. N. 2015/0018234, Rif. 63/12). All subjects gave written informed consent in accordance with the Declaration of Helsinki.

### Set Up

**Figure 1:**
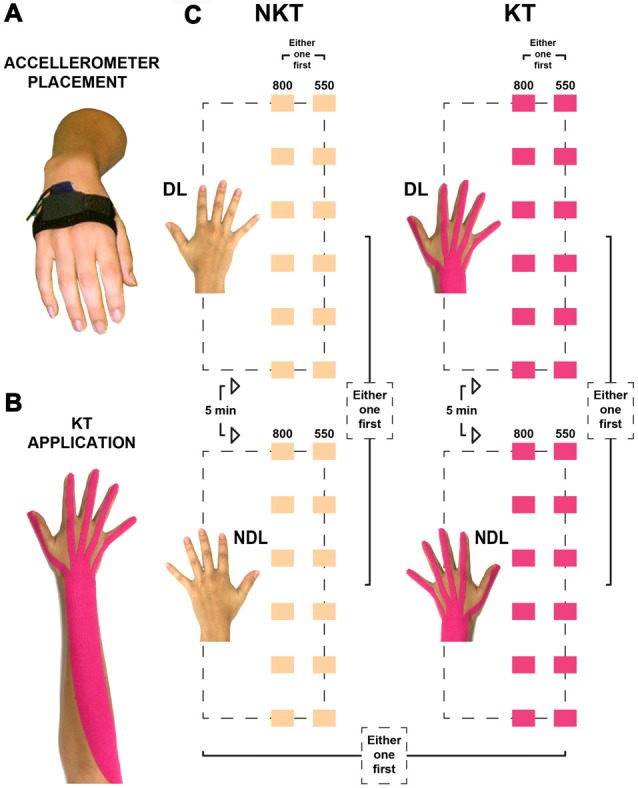
Placement of the accelerometer, application of the Kinesio^®^ Tex (KT) tape, and illustration of the two sessions. **(A)** The triaxial accelerometer was placed on the dorsal aspect of the hand, over the proximal part of the 2nd–3rd metacarpal bones, in a pocket kept in position by an elastic band and secured by a Velcro strap. **(B)** KT application on the wrist and fingers extensors from the lateral epicondyle of the humerus to the distal phalanges. **(C)** The no KT (NKT) and the KT sessions (color-coded in beige and pink, respectively) comprised each of a total of 24 IWFEs sets and were divided in two blocks. One block was performed exclusively with the dominant limb (DL), while the other one with the non-dominant limb (NDL). Each block consisted of 12 IWFEs sets performed in two conditions, six of them at 800-ms inter-onset interval (IOI), the other six at 550-ms IOI. The performance of the second block started after a 5-min rest interval from the end of the first block.

The set up is fully described elsewhere (Bravi et al., 2014a,b, 2015, 2016) and is summarized here. Every participant was tested individually, sitting upright on a chair with the feet on leg rest. Participant was asked to wear eye mask to prevent interference from visual information as well as headphones (K 240 Studio, AKG Acoustics GmbH, Wien, Austria) through which audio files could be heard. The forearms of participant were well placed on armrests of the chair, in a relaxed horizontal position. The wrist and hand were free to move in mid-air with no direct opposition, thus minimizing tactile information. The angle of the elbow joint was the result of the subject sitting in a comfortable position while respecting the prerequisites of maintaining the wrist and hand free to move without any possibility to touch, with any part of the hand, the armrest during the task. In any case, the elbow joint angle, measured by a goniometer, averaged around 100° (± 5°). A triaxial accelerometer (ADXL330, Analog Devices Inc., Norwood, MA, USA) was placed on the dorsal aspect of the hand when performing the rhythmic task. Triaxial accelerometer was sited over the proximal part of the 2nd–3rd metacarpal bones (Figure 1A). Sensor output was acquired and digitized at 200 Hz through PCI-6071E (12-Bit E Series Multifunction DAQ, National Instruments, Austin, TX, USA). Streams of clicks were generated by using Audacity^®^, via the Generate Click Track function. Each sequence contained 16 clicks with constant IOIs of 550- and 800-ms. Each click sound of 20 ms duration (set to white noise) was followed by 530-ms of silence for the IOI of 550-ms or by 780-ms of silence for the IOI of 800-ms.

### KT Application

The KT tape (Kinesio Holding Company, Albuquerque, NM, USA) is comprised of a polymer elastic strand wrapped by 100% cotton fibers and waterborne acrylic pressure-sensitive adhesives. KT is applied to the paper substrate with approximately 25% of tension, and the adhesive is 100% acrylic (Kase et al., 2013). Following the previously described protocols (Bravi et al., 2014b, 2016), KT application was designed with the purpose of covering the open kinetic chain including wrist, metacarpal and finger joints (Figure 1B). In order to accomplish this goal, the participant, already seated on the chair with forearm in full pronation and rested on the armrest, was asked to keep the wrist in full flexion. After manually assessing the origin (i.e., lateral epicondyle) and insertion (i.e., distal phalanges) of extensors muscles of the kinetic chain as a whole, the distance between the lateral epicondyle of the humerus and the distal end of the third phalanx of the middle finger was measured with a tape meter. The strip of KT was cut 5 cm longer than the maximum length of the measured kinetic chain (Kase et al., 2013). The course of the tendons of the extensor muscles (for each finger) was then identified on the back of participant’s hand and distances were measured between the distal end of each phalanx and the wrist. Measurements were used to cut the distal side of the elastic band into five branches to be placed over the metacarpal area and fingers following the course of the tendons. Once the tape was cut to the desired configuration, KT was applied from origin to insertion of wrist and fingers extensors of the arm. Specifically, KT was applied from the lateral epicondyle of the humerus to metacarpal area and fingers with moderate length tension (50% of the maximum available tension). In order to identify the percentage of KT tension, we have considered the length of KT when the tape is off the paper (expressed in cm) as a reference point (0%). KT was stretched to its maximum available tension. During application, since the technique required a length tension of 50%, this would translate to 50% of the difference (expressed in cm) between the maximum available length and the reference point length. KT was applied to all participants by the same investigator to ensure consistency throughout the study (Bravi et al., 2016). This procedure was repeated two times in order to apply the strip of KT on the wrist and fingers extensors of both the dominant and non-dominant arm, respectively.

### Sessions

**Figure 2:**
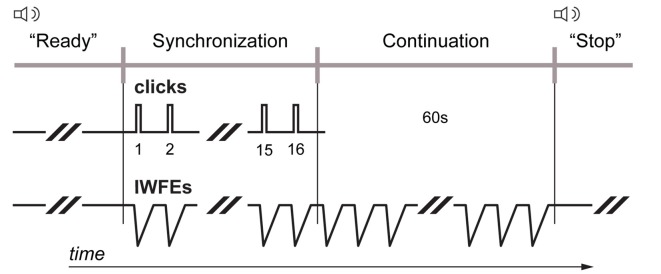
Synchronization-continuation task. Each participant was required to entrain isochronous wrist’s flexion-extensions (IWFEs) to the paced clicks so that the wrist flexion peak would coincide with the presentation of discrete auditory event (synchronization phase). When the stream of clicks ceased, participants continued to produce movements with the same temporal interval for 1 min until a vocal stop signal was given by experimenter announcing the end of the set of IWFEs (continuation phase). Streams of paced audio stimuli had an IOI of 550 and 800 ms, respectively.

All individuals had participated in two sessions, one with no KT (the NKT case) and one with KT application (the KT case; Figure 1C). Sessions were performed at least at a 3 days’ distance (Bravi et al., 2014b, 2016). Since it was found that the time of the day affects people’s timing performance (Lotze et al., 1999), participants executed the sessions during daylight hours, with each participant performing the two sessions systematically at the same hour of the day. The order of the two sessions was randomized between participants. In the KT session the test started after having applied the strip of KT on both dominant and non-dominant arm, starting with the application of KT on the arm by which the sets of IWFEs would be performed first. The synchronization-continuation paradigm was adopted for this experiment (Repp and Steinman, 2010; Braun Janzen et al., 2014). Each participant was asked to entrain IWFEs to the clicks so that the point of wrist flexion peak would coincide with the presentation of discrete auditory event (synchronization phase). When the stream of clicks ceased, participants continued to produce movements with the same temporal interval for 1 min until a vocal stop signal was given by experimenter announcing the end of the set of IWFEs (continuation phase; Figure 2). The duration of the continuation phase in each set of IWFEs was controlled by a stopwatch.

Each session began with instructions on the rhythmic motor task to complete as well as on how the sets of wrist’s flexion-extensions would be performed (for criteria see “Set Up” Section). This phase was followed by a short practice test to familiarize the participant with the task. Before data collection started, it was assessed whether the instructions were understood and whether the participant felt comfortable with the task. Two blocks of synchronization-continuations composed each session. One block was performed exclusively with the DL (Figure 1C), while the other one with the NDL (Figure 1C). Each block consisted of 12 IWFEs sets, six for each of the two IOIs (550- and 800-ms) conditions. A whole set of IWFEs lasted approximately 68.8 s in 550-ms IOI condition or 72.8 s in 800-ms IOI condition. The passage from the first block to the second one was after a 5-min rest interval. The order of blocks and IOI conditions was randomized to obtain a balanced number of subjects that executed sets of IWFEs with first one hand or the other and received first one IOI or the other. A set began when the experimenter asked whether the participant was “ready” after which the stream of clicks engaged and the participant moved in synchrony with it. A 60-s rest interval separated each set of IWFEs to avoid fatigue during performance (Bonassi et al., 2016; Figure 1C).

### Data Format and Statistical Analysis

Kinematic parameters were evaluated from a total of 1200 sets of movements (48 sets per participant). Data from the accelerometer were stored on a computer and an off-line analysis was implemented. The signal extracted from the accelerometer presented a minimum when the wrist reached the maximum flexion and a maximum when it reached the maximum extension. The duration of a single wrist’s flexion-extension (i.e., IWFE duration) was calculated as the distance between two consecutive flexion-extension minima (custom software developed in Matlab^®^). Only the data from the continuation phase were analyzed since the synchronization phase was used only to induce the desired frequency of IWFEs. In addition, since changes in timing were commonly observed at the transition from the synchronization to continuation phase (Flach, 2005), the first 5 s of the continuation phase in each recording were excluded from analysis. The last IWFE before or across the vocal stop signal was also excluded.

To assess the effect of the KT and the limb on the observed IWFEs durations, we adopted a random effect analysis of variance (ANOVA) model for repeated measurements (Pinheiro and Bates, 2000; Diggle et al., 2002), as previously used in Bravi et al. (2014b).

Two separate random effect ANOVA models were performed on data collected in the 550-ms and 800-ms IOI conditions. The response variable was the difference between the observed IWFEs duration and the expected duration in each condition. In the following this variable will be called error duration. The explanatory factors were the KT (presence/absence) and the limb (dominant/non-dominant).

The random effect ANOVA model adopted for the analyses has parameters that can be partitioned into two parts: the fixed effect part and the random effect part. The fixed effect parameters model the average response as dependent on the explanatory factors and their interaction as an ordinary ANOVA model. We used a dummy coding for the factors in the fixed effect part. We set NKT = 0 and KT = 1, DL = 0 and NDL = 1. In addition, as the response variables have been recorded several times for each performance and for each individual, random effect parameters had to be included to take into account the lack of independence among the observations. The random effect part was specified in order to separately measure the variability within individuals and within performances. In particular, we adopted a random effect ANOVA model with both a random intercept and a random slope, in which the random effect variability (measured by standard deviation (SD)) depends on the explanatory factors and their interaction. This model takes into account for possible residual heteroscedasticity. A lower random effect residual SD reflects a stronger proficiency in the production of the IWFEs durations.

Specifically, the model has three levels of variation. The three levels correspond to: (1) single IWFEs duration on which error duration is measured; (2) series of IWFEs durations as sets of movements (48 sets per participant); and (3) individuals, performing the 48 sets of IWFEs durations. At the IWFEs duration level (1), within each set, we adopted an autoregressive AR(1) model for the random effects. The covariance between errors in duration *i* and duration *j* in set *k* is the variance in set *k* times *ρ*^|i-j|^ where *|i-j|* is the absolute value and *ρ* is the parameter measuring the correlation between two subsequent durations. At the set of IWFEs duration level (2), the random effects have different variances for each combination of treatments (NDL-NKT, NDL-KT, DL-NKT and DL-KT). The combination NDL-NKT has been considered as baseline category. The SD for the combination *h* (*h* = NDL-KT, DL-NKT or DL-KT) have been parametrized as: SD_h_ = baseline SD * ratio *h*. Individuals have been considered independent, with constant variance.

In order to display the effect of KT on the variability of the timed performance of the single individual, we performed Poincaré maps or return maps of the time series of IWFEs durations (Shenker, 1982; Mendez-Balbuena et al., 2012). A Return map is a graph of the IWFEs duration *x*_i+1_ vs. the previous IWFEs duration *x*_i_ where *i* is the actual observation. According to the return maps a timed performance with lower timing variability will have a smaller dispersion of the points in the graph.

In addition, we investigated if KT influences the short-term dependencies in the sets of IWFEs durations by computing lag-one autocorrelation—*ρ*(1)—analysis. *ρ*(1) is the autocorrelation of a series with itself, shifted by a particular lag of 1 observation. Positive *ρ*(1) describes a series in which adjacent observations move generally in the same direction. In the presence of a strong linear trend, it would be expected a value of *ρ*(1) close to 1. Conversely, negative *ρ*(1) reflects swings in the set, in which high values are immediately followed by low values and vice versa (Dunn, 2005). The presence of a slow natural change in tempo resulting in differing expected tap intervals at different points in time (drift) is occurring in time long interval sets (Collier and Ogden, 2004). Since the presence of drift’s behavior in time interval series could be a source of positive autocorrelations in long series in continuation time sets (Collier and Ogden, 2004), we performed a series of detrended windowed lag-one autocorrelations, herein abbreviated w*ρ*(1), for each set of IWFEs (Lemoine and Delignières, 2009; Bravi et al., 2014b). We computed w*ρ*(1) over a window of the 30 first points, moving the window by one point, all along the sets. To analyze the observed w*ρ*(1) we adopted random effect ANOVA model for repeated measurements. In order to allow an appropriate use of parametric statistical tests, the Fisher’s Z-transformation was used to normalize the distribution of w*ρ*(1) (Nolte et al., 2004; Freyer et al., 2012).

The significance level was set at *p* ≤ 0.05 for the analyses in the fixed effect part of the random effect ANOVA model for repeated measurements.

## Results

To assess the effect of the KT and the limb on the observed IWFE durations, we performed random effect ANOVA models separately for the 800-ms and 550-ms IOI conditions. The estimates of the error duration for the fixed effect part of the models in the two IOI conditions are reported in Table 1, together with their *p*-values, *t*-values and confidence intervals. As already mentioned in “Data Format and Statistical Analysis” Section, for these models the error duration is the difference between the observed and the expected IWFEs duration (800-ms and 550-ms, respectively).

**Table 1:** Estimates of model parameters of the random effect ANOVA model for the isochronous wrist’s flexion-extensions (IWFEs) durations, *p*-values (in parenthesis), *t*-values and confidence intervals for the 800- and 550-ms inter-onset interval (IOI) conditions.

Main fixed effect	800-ms IOI			550-ms IOI		
	Estimate (*p*-value)	*t*-value	95% confidence interval	Estimate (*p*-value)	*t*-value	95% confidence interval
Intercept	−16.2 (0.0366)	−2.0905	−31.3; −1.0	1.8 (0.6872)	0.4027	−6.8; 10.4
Non-dominant limb	−8.9 (0.0027)	−3.0129	−14.6; −3.1	0.9 (0.5754)	−0.5604	−3.9; 2.2
With KT	5.9 (0.0438)	2.0203	0.2; 11.7	−12.5 (0.0000)	−8.0448	−15.5; −9.4
**Random effect**	**Estimate**		**95% confidence interval**	**Estimate**		**95% confidence interval**
Within-individual SD	36.5		27.4; 49.1	20.9		15.4; 28.2
Within-set SD	35.6		33.5; 37.7	18.7		17.7; 19.9
*ρ* (AR1)	0.24		0.23; 0.25	0.38		0.37; 0.39
NDL-NKT/NDL-KT residual SD ratio	0.992		0.976; 1.009	0.851		0.837; 0.865
NDL-NKT/DL-NKT residual SD ratio	1.069		1.051; 1.087	0.937		0.918; 0.957
NDL-NKT/DL-KT residual SD ratio	1.035		1.018; 1.052	0.850		0.836; 0.863

For the 800-ms IOI condition, the intercept, that is the estimate of error duration when movements were performed with DL and without KT, was found to be negative and significant (i.e.,−16.2 ms; *p*-value = 0.0366; Table 1), indicating that observed IWFEs durations were, on mean, shorter than those expected. The effect of NDL was highly significant on influencing the error duration, which was found to be negative (i.e.,−8.9 ms; *p*-value = 0.0027). This implies that IWFEs durations produced with the NDL were on average about 9 ms shorter than those achieved with the DL. The application of KT on the DL (with KT; Table 1) corrected significantly toward the expected IWFEs durations (5.9 ms; *p-value = 0.0438*). The interaction between the NDL and the KT was not found significant (estimate: 3.1 ms; *p*-value = 0.5984) indicating that the effect of KT in modeling the error duration does not vary with the limb on which it is applied. For the 550-ms IOI condition, the intercept was not found to be significant (i.e., 1.8 ms; *p*-value = 0.6872; Table 1) as well as the effect of NDL (i.e., 0.9 ms; *p*-value = 0.5754). Consequently, participants were on average slower and almost equally accurate at producing the expected IWFEs durations with dominant or NDL when KT was not applied. Conversely, KT, when applied on the DL (with KT), had a highly significant effect on the error duration by shortening IWFEs durations (i.e.,−12.5 ms; *p-value* = 0.0000). Also here the interaction between the NDL and the KT was not found significant (estimate: 0.2 ms; *p-value* = 0.9378).

Additionally, random effect residual SD estimates were computed for each condition. The ratio of residual SDs estimates was analyzed to determine if there were significant differences among cases. Coding the effect of a factor on a SD via a ratio guarantees the derived SD to be positive. The residual SD for NDL-NKT case was considered as baseline (residual SD = 1.00) and compared with residual SD for the other three cases (NDL-KT, DL-NKT and DL-KT). A SD ratio lower or greater than one means that timing variability of a specific case is reduced or augmented respect to the baseline (NDL-NKT) case. Two conditions are considered significantly different when their confidence intervals do not overlap (for details on random effect model for heterogeneous population, see Muthén, 1989). Estimates and confidence intervals resulting from ratio between the residual SD for NDL-NKT and residual SDs for other three NDL-KT, DL-NKT and DL-KT cases are given in Table 1 (see Random effect part).

**Figure 3:**
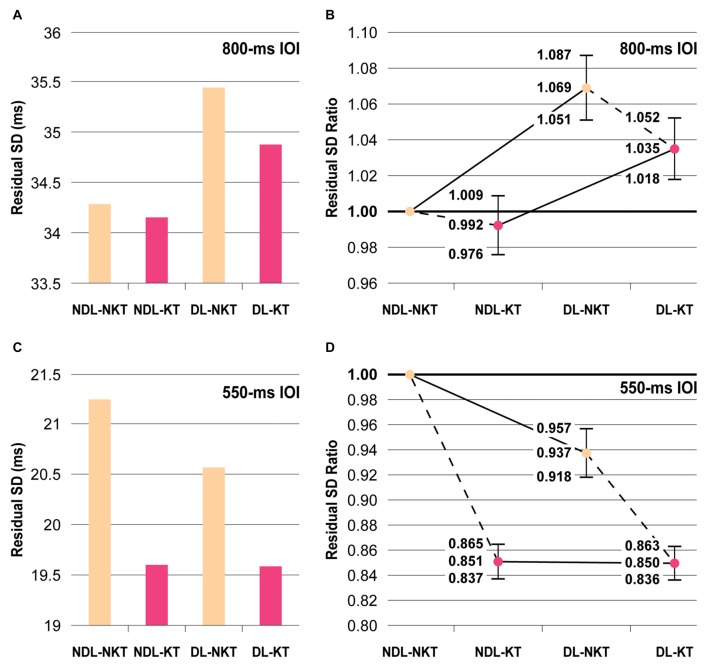
Mean residual SDs of IWFEs durations and residual standard deviation (SD) ratios. **(A,B)** In the NKT cases, participants are more precise in performing slow rhythmic movements (800-ms IOI) by using NDL. The NDL proficiency is maintained also in the KT (color-coded in pink) cases. **(C,D)** In the NKT (color-coded in beige) cases, subjects were more precise to perform faster rhythmic movements (550-ms IOI) with the DL. The application of KT counterbalances the between-hand differences in performance. Panels **(B,D)** show the estimates and confidence intervals (vertical bars) resulting from the ratio between the residual SD for NDL-NKT and the residual SDs for other three NDL-KT, DL-NKT and DL-KT cases.

For the 800-ms IOI, the residual SD was found to be 34.29 ms in NDL-NKT case, 34.15 ms in NDL-KT case, 35.45 ms in DL-NKT case, and 34.88 ms in DL-KT case (Figure 3A). According to the confidence intervals of the SDs ratio over the experimental conditions, KT reduced the variability of IWFEs performed with the dominant or the NDL, but in both cases such decrease did not reach the significance level (i.e., NDL-NKT vs. NDL-KT or DL-NKT vs. DL-KT; Figure 3B). Conversely, significant differences were shown in the NKT cases when comparing the non-dominant and the DL (i.e., NDL-NKT vs. DL-NKT; Figure 3B). Individuals were found to be more precise in performing slow rhythmic movements by using the NDL (Figures 3A,B). Significant differences were also maintained in the KT cases when comparing the non-dominant and the DL (i.e., NDL-KT vs. DL-KT; Figure 3B).

For the 550-ms IOI, the residual SDs of all cases showed, in general, values smaller than those achieved in the 800-ms IOI condition. The residual SD was found to be 21.25 ms in NDL-NKT, 19.60 ms in NDL-KT, 20.57 ms in DL-NKT, and 19.59 ms in DL-KT cases (Figure 3C). According to the confidence intervals of the SDs ratio over the experimental conditions, significant differences were shown in the NKT cases when comparing the non-dominant and the DL (i.e., NDL-NKT vs. DL-NKT; Figure 3D). However, differently from that observed in the 800-ms IOI condition, subjects were more precise in performing IWFEs with the DL (Figures 3C,D). Also, significant differences were found between the NDL-NKT and the NDL-KT cases, or the DL-NKT and the DL-KT cases, respectively (Figure 3D). The application of KT, unlike to 800-ms IOI, helped to decrease significantly in both limbs the timing variability of IWFEs durations (Figures 3C,D), suggesting that the effect of KT is influenced by the frequency of movement being performed. Finally, the NDL-KT and the DL-NKT cases presented significant differences (Figure 3D), showing that KT, when applied on NDL, differently from what happens for the slower movements, not only counteracts the precision disadvantage respect to the DL but it makes the NDL more precise than the dominant one. These significant differences were lost when KT was applied on DL (i.e., NDL-KT vs. DL-KT; Figures 3C,D).

**Figure 4:**
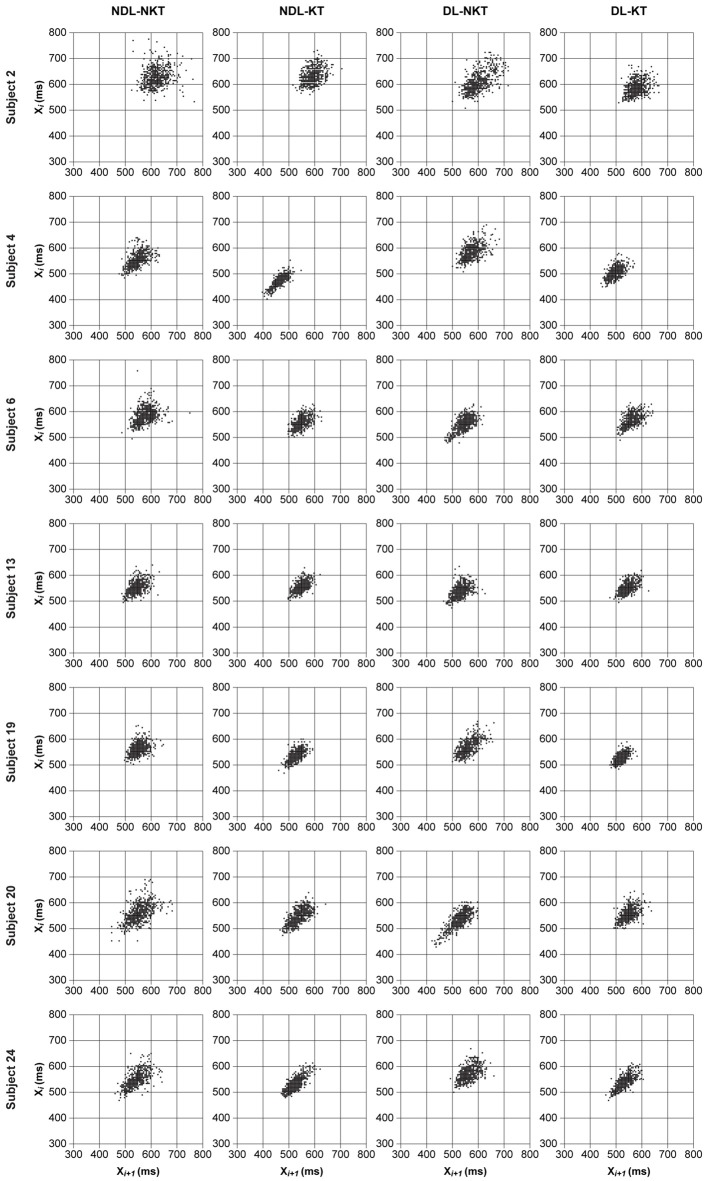
Return maps of the IWFEs durations for NDL-NKT, NDL-KT, DL-NKT and DL-KT in the 550-ms IOI condition. Each return map illustrates, per subject, durations of IWFEs for all six sets performed. A smaller dispersion of all points in the graph indicates a reduction of timing variability. The seven subjects were chosen to show slightly different behaviors. It is noticeable that the dispersion of points in KT cases is smaller than in NKT cases.

To visualize the effect of KT on reducing timing variability of IWFEs durations performed at IOI of 550-ms, we used the qualitative analysis of the Poincaré maps or return maps. Figure 4 displays return maps of seven subjects in the four conditions: NDL-NKT, NDL-KT, DL-NKT and DL-KT. The dispersions of the points in the maps, per subject, are smaller in the KT cases than those in the NKT cases, meaning that in the KT cases the IWFEs were performed more proficiently than in the NKT cases in which a large dispersion of the points is shown.

In addition, we explored whether and to what extent KT and the limb modulated the short-term dependencies in the sets of IWFEs durations by studying the w*ρ*(1). We adopted random effect ANOVA models separately for the 800-ms and 550-ms IOI conditions. The estimates of the w*ρ*(1) of the IWFEs durations for the fixed effect parameters in the two IOI conditions are reported in Table 2, together with their *p-values*, *t*-values and confidence intervals. For the 800-ms IOI condition, the intercept, that is the estimate of the w*ρ*(1) of IWFEs durations when movements were performed with DL and without KT, was found to be positive and significantly different from the value 0 (i.e., 0.180; *p*-value = 0.0000; Table 2). The NDL had no significant effect on w*ρ*(1) (i.e., 0.024; *p-value = 0.3078*). In addition, KT, when applied on the DL (with KT; Table 2), did not modulate significantly the w*ρ*(1) of IWFEs durations (i.e.,−0.028; *p* -value = 0.2228), and the interaction between NDL and KT was not found to be significant (estimate: 0.012; *p*-value = 0.7179). These results suggest that KT when applied on dominant and NDL does not influence the short-term dependencies in the sets of IWFEs durations performed at IOI of 800-ms.

**Table 2:** Estimates of model parameters of the random effect ANOVA model for the w*ρ*(1) of IWFEs durations, *p*-values (in parenthesis), *t*-values and confidence intervals for the 800- and 550-ms IOI conditions.

Main fixed effect	800-ms IOI			550-ms IOI		
	Estimate (*p*-value)	*t*-value	95% confidence interval	Estimate (*p*-value)	*t*-value	95% confidence interval
Intercept	0.180 (0.0000)	5.8333	0.119; 0.236	0.316 (0.0000)	12.2551	0.270; 0.368
Non-dominant Limb	0.024 (0.3078)	1.0208	−0.003; 0.062	−0.014 (0.4213)	−0.8047	−0.045; 0.005
With KT	−0.028 (0.2228)	−1.2205	−0.054; 0.010	0.066 (0.0002)	3.7592	0.036; 0.085
**Random effect**	**Estimate**		**95% confidence interval **	**Estimate**		**95% confidence interval**
Within-individual SD	0.131		0.097; 0.178	0.113		0.084; 0.152
Within-set SD	0.199		0.187; 0.211	0.152		0.143; 0.161

Higher positive values of w*ρ*(1) were found in the 550-ms IOI condition respect to the 800-ms IOI condition (Table 2). The intercept was found to be positive and significantly different from the value 0 (i.e., 0.316; *p*-value = 0.0000; Table 2). The NDL did not influence significantly the w*ρ*(1) of IWFEs durations (i.e.,−0.014; *p*-value = 0.4213). Differently from what happens for the slower movements at 800-ms IOI, the effect of KT, when it was applied on the DL, on the w*ρ*(1) was highly significant and positive (i.e., 0.066; *p-value* = 0.0002). Also in this case, the interaction between the NDL and KT was not found to be significant (estimate: −0.011; *p*-value = 0.6448). Overall, the application of KT influences significantly w*ρ*(1) having the IOI of 550-ms and this effect does not vary with the limb on which it is applied.

## Discussion

The results show that timing precision asymmetries between dominant and non-dominant hands are present when IWFEs are performed at the two different frequencies investigated. Work by Peters (1976), although on a single subject, showed a difference between sides for finger tapping executed at maximal rate in terms of timing variability of intertap intervals, with the DL performing more regularly than the NDL. Todor et al. (1982) showed that side differences, in rate and variability, of tapping exist not only for distal joints (i.e., finger), but also when the movements are performed at more proximal joints (i.e., wrist and shoulder). In addition, Schmidt et al. (2000) demonstrated that asymmetry for intertap variability is significantly greater in right-handers than left-handers when performing with the DL. Also, they confirmed the earlier observations relative to DL superiority in execution of rhythmic movements with higher precision when the performance is requiring maximal speed (Peters, 1976; Todor et al., 1982; Schmidt et al., 2000). According to this literature, and shown also by our findings, there seems to be a precision advantage of the DL which is reflected by a smaller timing variability, when IWFEs are performed at faster rates (550 ms-IOI). However, this precision-based DL superiority is lost when IWFEs are performed at slower rates (800 ms-IOI). The opposite is true for the NDL, with a more precise performance, compared to the DL, at slower rates. These findings may suggest different preferred temporal environments, specific to the dominant and non-dominant motor effectors, when performing sequential motor actions.

At present, we can only speculate on reasons for this phenomenon. One possibility is that the mode of temporal processing for motor control (Peters, 1976; Todor and Kyprie, 1980) between the two arms is different, being dependent on exploitation of different sensorimotor processes and neuromuscular resources that each arm had strengthened for the execution of functional habitually movements. It was suggested that for sequential rhythmic actions of supra-second durations, a more cognitive control is employed. For sub-second durations however, the circuitry used to ensure the consistency of rhythmic movements is assumed to be ingrained more tightly within the motor system (Lewis and Miall, 2003). This hypothesis resides in the fact that voluntary movements are typically of sub-second durations and can be reproduced with extreme temporal precision (Lewis and Miall, 2003). Recently, it was shown that also cognitive control processes might influence sub-second repetitive motor timing actions (Holm et al., 2017). Optimal control of goal-directed arm movements is proposed to reflect two strategies, feedforward and feedback control (Kawato, 1999; Shadmehr et al., 2010). Feedback and feedforward sensorimotor control of human movements, rather than working independently, complement each other to guarantee motor performance with a high precision (Gritsenko et al., 2009; Ao et al., 2015). Also, it was shown that control strategies during voluntary goal-directed movements are influenced by speed, shifting from feedback to feedforward control as the speed increases (Kawato, 1999; Gerisch et al., 2013; Ao et al., 2015).

Furthermore, the most leading theories attempting to describe the neurophysiological basis of interlimb performance differences are the so-called open vs. closed loop and the dynamic dominance. The former speculates that arm differences are derived from specialization of dominant and non-dominant systems for different mechanisms for motor control: dominant system for feedforward processes and non-dominant system for sensory feedback mediated error correction mechanisms (Haaland and Harrington, 1994; Hermsdörfer et al., 1999). The second hypothesizes that the dominant arm, by relying on a predictive dynamic control, is specialized for optimizing dynamic features of movement whereas the non-dominant arm, by employing a feedback- and impedance-based positional control mechanisms, is specialized in stabilizing tasks and corrective movements (Bagesteiro and Sainburg, 2002; Mutha et al., 2013). According to the open vs. closed loop theory, the differences between dominant and non-dominant side that we found for movements with temporal durations of 550- and 800-ms could reflect the different specialization of each arm for the employment of specific different mechanisms for motor control. In particular, we speculate that, for fast rhythmic movements, a better proficiency of the dominant arm in relying on feedforward processes could favor the reduction of variability of temporal movements; viceversa, below a certain threshold of speed, there is a greater dependency on feedback processes and, consequently, the non-dominant arm, by being more feedback dependent, will produce a better performance.

**Figure 5:**
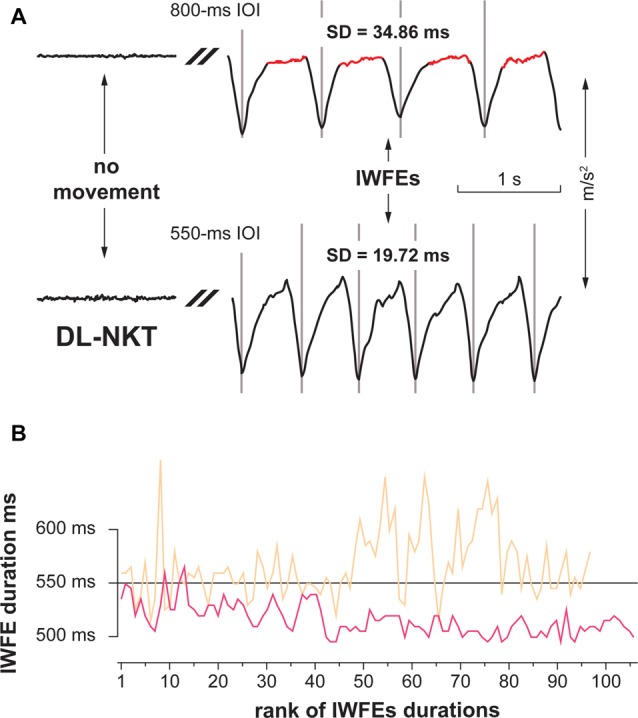
Examples of kinematic parameters of two representative short sequences of IWFEs (**A**, upper panel), and representative sets of IWFEs durations in the NKT and KT case (**B**, lower panel). **(A)** IWFEs performed, with the DL and without KT, by a participant in the 800- and the 550-ms IOI conditions. The baseline tracing of the recording, where there is no movement, is illustrated on the left side of the Figure. The trace is cut since only IWFEs pertaining to the continuation phase of the recording are shown. A gray vertical line marks the onset of each IWFE. The duration of a single IWFE is the distance between two consecutive flexion-extension minima. It is possible to observe that movements in the 800-ms IOI condition (upper trace) are characterized by a pause after each downstroke (marked as red), while movements in the 550-ms IOI condition (lower trace) are performed in a rather continuous way. SDs of IWFEs durations for the corresponding sets are also given. **(B)** In the lower panel, sets of IWFEs durations performed by a participant with the DL without KT (color-coded in beige) and with KT (color-coded in pink). Note that the variability of IWFEs durations is remarkably reduced when KT is applied. Also, it is illustrated that the reduced variability of IWFEs durations when KT was applied is associated with the tendency of IWFEs durations to decrease during performance.

On the other hand, when considering the dynamic dominance theory, the speed of movement is critical in influencing the shape of rhythmic actions (Huys et al., 2008; Repp, 2008). Rhythmic movements performed in a natural way (i.e., with no specific indication) at a slow pace were shown to have a discrete shape (i.e., characterized by singularly occurring events preceded and followed by periods of stabilizing posture in absence of motion), whereas fast movements were demonstrated to possess a continuous configuration (Huys et al., 2008). In Figure 5A are shown two typical examples of kinematic parameters of sequences of movements performed with the DL by a participant in the 800- and the 550-ms IOI conditions. It is possible to observe that movements in the 800-ms IOI condition are characterized by a pause after each downstroke, while movements in the 550-ms IOI condition are performed in a rather continuous way. Therefore, in agreement with the dynamic dominance theory, it is also possible that the NDL, by engaging a feedback- and impedance-based positional control mechanisms, could perform more proficiently than dominant hand in a rhythmic task, like the 800-ms IOI condition, in which stabilizing postures and dynamic movements are both present. Conversely, a rhythmic task of 550-ms IOI, in which the dynamic features of movement are preponderant, could be a condition particularly fitting for the DL due to a greater proficiency in employing predictive dynamic mechanisms.

Another possibility is that participants, by perceiving slower IWFEs less stable when performed by non-dominant hand, are trying to compensate for it by allocating more attention for the execution of isochronous motor actions. However, this alternative hypothesis seems to be unlikely due to evidence showing that increased cognitive load (i.e., working memory and executive load) influences variability of the rhythmic motor performance, by increasing it (Holm et al., 2013, 2017; Bravi et al., 2014a). A recent brilliant study by Holm et al. (2017) tested the influence of executive functions in repetitive motor timing by using a synchronization-continuation task. In this study participants were asked to repeat a fixed three finger sequence (low executive load) or a pseudorandom sequence (high executive load) executed at different tempi. It was shown that, while not for the longer IOIs (1024-ms and 1431-ms IOIs), high load increased timing variability for 524-ms and 733-ms IOIs. Therefore, data available in literature reinforce our hypothesis that the precision asymmetry between hands, here demonstrated as preferred temporal environment, could reflect limb differences in exploitation of different sensorimotor processes for the production of movement. Whatever the case may be, our results add a new piece of information to the context of motor timing literature, revealing that hand advantages/preferences in performing proficiently rhythmic movements are not side-locked but depend also on the speed of movement.

The use of KT in our experiments is designed to add some sensorial feedback through a wearable device able to influence proprioceptive information and modify performance precision. Our results, besides confirming previous data, show that KT improves the consistency of IWFEs (Bravi et al., 2014b, 2016). However, the frequency in which IWFEs are performed is crucial to determine the extent of the KT effect. We found that, while KT, on average, reduced significantly timing variability of 550 ms (1.81 Hz) IWFEs, it was not able to improve consistency of IWFEs having a duration of 800 ms (1.25 Hz). In addition, the effect of KT was hand-independent. In Figure 5B are illustrated sets of IWFEs durations performed by a participant with the DL without and with KT. It is possible to note the remarkable reduction of variability of IWFEs durations when KT is applied. We ascribe the observed KT effect in the 550-ms IOI condition to an extra-proprioceptive information provided by KT application. In fact, KT was shown to influence significantly proprioception (Pelosin et al., 2013; Seo et al., 2016). Also, somatosensory feedback was shown to be critical in influencing the precision of the variability of movements in tasks of timing coordination (LaRue et al., 1995; Spencer et al., 2003; Bravi et al., 2014b). Specifically, we speculate that KT due to its elastic properties, during the phase of wrist flexion, applies a pulling force that, in turn, provides an additional stimulation of cutaneous, and presumably muscle, mechanoreceptors by stretching and deforming the skin as well as targeting deeper muscle tissues (Bravi et al., 2014b, 2016; Pamuk and Yucesoy, 2015). Such effect of KT would augment the coordination of the wrist joint during the rhythmic motor performance and, consequently, contribute to the reduction in timing variability of the IWFEs (Bravi et al., 2014b). The extra-proprioceptive hypothesis is plausible since in our experimental paradigm IWFEs were performed with no direct surface opposition and while minimizing visual information, thus accentuating the role of the sensory component that provides limb position and movement senses to produce rhythmic actions as accurate as possible (Guigon et al., 2008; Bravi et al., 2014a,b, 2016).

In Bravi et al. (2016), we investigated whether different directions and tensions of KT application differently influenced the precision of sensorimotor synchronization. We showed a highly significant effect of KT in improving the precision of the performance of IWFEs having duration of 500 and 400 ms (2 and 2.5 Hz, respectively). Therefore, if data obtained previously (Bravi et al., 2016) and in this study are compared it might be possible to locate a time region of optimal adaptability of the motor output to the sensory information provided by KT. It seems that KT does manage more efficiently the rhythmic behavior within specific temporal windows, in which a control mechanism has been hypothesized to operate in an optimal, or preferential, state of activity for the production of rhythmic motor behaviors (McAuley et al., 2006). This preferential state is identified as spontaneous motor tempo, that is, a preferred rate in which rhythmic actions are performed. Although each individual has its own spontaneous tempo, rhythmic motor actions in humans were shown to be performed, on average, with a preference/spontaneous frequency of around 2 Hz. Locomotion studies conducted both in laboratory and natural settings showed a highly tuned resonant frequency of human locomotion at 2 Hz (Murray et al., 1964; MacDougall and Moore, 2005). A predilection for a 2-Hz frequency of movement has also been observed in subjects freely tapping out a rhythm (Collyer et al., 1994; Vanneste et al., 2001; Bisio et al., 2015). Collyer et al. (1994) reported a bimodal distribution of spontaneous motor tempi in which the main of these was around 2.2 Hz (equivalent to 450 ms duration), while McAuley et al. (2006) showed that spontaneous motor tempo was influenced across the life span, and that adults of ages ranging between 18 and 38 (very similar to age of group in our study) preferred to perform rhythmic movements with an interval duration of 1.66 Hz (equivalent to 600 ms interval duration).

Moreover, it is speculated that the spontaneous motor tempo reflects the intrinsic rate of a spinal central generator (MacDougall and Moore, 2005). Evidences suggest that in humans both the arms and legs are regulated by CPGs and that sensory feedback contributes strongly to the modulation of the putative CPG output (Van de Crommert et al., 1998; Marder, 2001; Harischandra et al., 2011) and assists in mediating interlimb coordination (Zehr and Duysens, 2004). Kuo (2002), by using a model of a single pendulum driven to oscillate in a manner analogous to limb motion, explored how feedforward and feedback can be combined to control rhythmic limb movements. He demonstrated that a cooperation of these mechanisms could improve performance in systems subject to both unexpected disturbances and sensor noise. In this model, a CPG acts as an internal model by making a sensory prediction of limb movement that, in turn, drives the activation of the feedback mechanism. During motion, the magnitude of incongruity between the commanded and the occurring movement results in sensory error signals that are fed back to the oscillator, which entrains a feedforward component to the actual movement (Kuo, 2002). The adjustment of the expected state is used to produce the appropriate feedback command. However, sensory information provided by proprioceptors is not perfectly accurate as that of the pendulum model, and such uncertainty, coupled with motor noise, directly translates into performance variability (van Beers et al., 2002; Guigon et al., 2008). Therefore, consistent with this model, changes in sensory signal provided by application of KT during movement, could reduce performance variability of IWFEs by compensating for such discrepancy between the commanded and the occurring movement that, in turn, would favor the generation of the appropriate feedback command for an augmented motor performance.

Additionally, we performed a detrended windowed lag-one autocorrelation analysis and we found positive values of w*ρ*(1) in both the IOIs conditions. In line with previous studies (Huys et al., 2008; Repp and Steinman, 2010; Bravi et al., 2014b, 2015), the highest w*ρ*(1) values were yielded for fast IWFEs. When NKT and KT cases were compared, we found that KT influences the short-term dependencies of IWFEs durations. Interestingly, KT biased w*ρ*(1) values of IWFEs towards higher positive values in the 550-ms IOI condition, but not in the 800-ms IOI. Also, our participants performed IWFEs faster compared to the expected interval durations (i.e., 550-ms IOI; see Table 1). Ivry and Keele (1989) reported that their trials showed a positive lag-one covariance after detrending and that the mean intertap intervals were less than the target of 550 ms (Ivry and Keele, 1989). Together with their findings, our present data indicate that some acceleration and, thus, some residual drifting tempo, may persist even after linear detrending. Our w*ρ*(1) analysis (see Table 2, for 550-ms IOI) substantiate their remark of a “drift effect”, when KT is applied. To summarize, the application of KT, while enhancing precision of performance, seems, paradoxically, to be associated with loss of cognitive control (Holm et al., 2017) during the production of repetitive motor actions.

In Bravi et al. (2014b), it was demonstrated that the improvement of timing precision of IWFEs provided by KT was associated with a modulation of the timing processes. By providing extra proprioceptive information and stabilizing wrist joint, the production of IWFEs could become less dependent on central drive (Bravi et al., 2014b). It is believed that the potential for interference between areas of cerebral cortex increases with the degree in which these areas are activated (Kinsbourne and Hicks, 1978; Carroll et al., 2001). Therefore, an augmented activity of lower circuitry appointed to the optimization of sensorimotor behavior could allow, at least in part, the release of control from time-bearing higher centers including those for cognition (Fischer et al., 2016), which would allow a net augmentation of the motor control efficiency and, ultimately, an improvement of timing precision. By extending and partly revisiting the hypothesis proposed in Bravi et al. (2014b), the increased consistency of the rhythmic motor behavior following application of KT could be ascribed to a combined adaptation effects occurring at both lower-spinal and higher central sites handling the production of IWFEs.

Finally, our results can also be explained from another perspective, which is to be confirmed in future experiments. It was shown in recent studies that tactile-proprioceptive noise is capable of improving the stability in sensorimotor performance when appropriate amounts of noise are used (e.g., Mendez-Balbuena et al., 2012; Trenado et al., 2014). The augmented performance precision is speculated to be due to an increased stimulation of cutaneous mechanoreceptors, causing, via internal stochastic resonance, an enhancement in neuronal firing synchronization at spinal and cortical level (Manjarrez et al., 2002) and cortico-spinal level (Mendez-Balbuena et al., 2012). This neuronal firing synchronization was reflected in spinalcortical and corticospinal coherence. Higher corticospinal coherence has been shown to be associated with better motor performance (Baker, 2007; Kristeva et al., 2007; Pogosyan et al., 2009).

Therefore, similarly to the tactile noise, an enhancement of stimulation of cutaneous mechanoreceptors provided by KT could reduce IWFEs timing variability by increasing the coherence between spinal and cortical neurons activity within the somatosensory system. A similar increase in such spinal-cortical coherence was found in cats when a particular intensity of tactile noise was applied on the skin (Manjarrez et al., 2002). As shown by Fisher et al. (2002), sensory information from cutaneous receptors enhances oscillatory synchrony in the motor system. Therefore, KT could increase sensorimotor integration at cortical level, leading to a greater cortical motor synchrony and a stronger motor cortex drive to the muscles (Mendez-Balbuena et al., 2012). It would be interesting to examine in future studies the effect of the KT on the cortico muscular coherence during a synchronization-continuation task, and whether a combination between KT and tactile noise could provide further stimulation to cutaneous receptors in order to improve the efficiency of motor control for a better performance.

By studying dominant and non-dominant upper limbs, we evaluated the differential effect of KT in influencing a rhythmic motor behavior and in counteracting timing precision difference between limbs. Significant effect of KT application was observed only at 550-ms IOI, consequently we will focus on this condition. In the 550-ms IOI condition, participants not wearing KT show a reduced ability to perform IWFEs consistently with the NDL. The application of KT not only cancels this precision disadvantage but it makes the non-dominant hand even more precise than the dominant one without KT. KT augmented also timing skills of dominant hand but only enough to neutralize the gap created by KT on the non-dominant hand.

Research on the contribution of sensory input in influencing motor performance asymmetries between arms denotes, as mentioned above, a non-dominant left arm/right hemisphere “sensory dominance” for the utilization of proprioceptive feedback in right-handed individuals (Colley, 1984; Riolo-Quinn, 1991; Goble et al., 2006; Goble and Brown, 2007, 2010). Conversely, the dominant system is suggested to function in a feedforward fashion (Goble and Brown, 2007), relying more on visual feedback (Honda, 1982). This asymmetry between upper limbs to exploit proprioceptive feedback is speculated to stem from functional differences in the roles of the dominant and non-dominant hands during bimanual tasks (Han et al., 2013). For instance, early results by Roy and MacKenzie (1978), who investigated arm differences in the ability to match thumb and multi-joint arm positions after depriving the subjects of visual information, revealed a non-dominant arm advantage for matching end positions of the thumb, with no arm differences for multi-joint arm matching (Roy and MacKenzie, 1978). Later, Colley (1984) and Riolo-Quinn (1991) confirmed the presence of a non-dominant thumb advantage to accomplish proprioceptive-guided matches, and Kurian et al. (1989) demonstrated a non-dominant arm supremacy for accurately reproducing elbow angles. More recently, Goble et al. (2006) by using a memory-based proprioceptive matching task, in which participants were required to memorize limb position and match with the ipsilateral and the contralateral arm, showed a specialization of the right hemisphere/left arm for proprioceptive feedback processing that is either position- or dynamic position-related (Goble and Brown, 2007, 2010).

Although the lower level of timing precision of NDL can impact on the effect of KT, the superior sensitivity of the NDL to KT, able even to overturn the original between-hand asymmetries, could be explained by the specific proficiency of the NDL to use the extra-sensory information provided by KT to correct ongoing movement.

## Conclusion

The results from this study shed light on the working mechanism of KT in rhythmic movement around spontaneous tempo. It seems that the effect of KT is more pronounced for certain temporal intervals, and that these intervals are reminiscent to those encountered in human walking (MacDougall and Moore, 2005; Styns et al., 2007). As such, the implementation of KT as an added measure in rehabilitation protocols, where rhythmic movement is impaired, may prove to be efficient. Although further investigations of the effect of KT are needed, for example, analysis of goal-directed movements (Kuling et al., 2016), an additional application of the KT method could be coupled with motor protocols for rehabilitation in impairments of the non-dominant motor system to enhance the use of movement-related proprioceptive information (Goble and Brown, 2007). Finally, at the other end of the motor system, in individuals with peripheral neuropathy, a condition that is known to reduce asymmetries in inter-limb transfer (Pan and Van Gemmert, 2016), KT could be thought of as an effective mean to enhance the motor performance. These latter speculations remain to be confirmed or rejected by future experimentation.

## Author Contributions

RB: substantial contributions to the conception or design of the work, the acquisition, analysis and interpretation of data for the work; final approval of the version to be published. EJC: substantial contributions to the conception or design of the work and interpretation of data for the work; drafting the work or revising it critically for important intellectual content. AM: substantial contributions to the analysis; drafting the work or revising it critically for important intellectual content. AG: substantial contributions to the inferential statistics analysis; drafting the work or revising it critically for important intellectual content. DM: substantial contributions to the drafting the work or revising it critically for important intellectual content; agreement to be accountable for all aspects of the work in ensuring that questions related to the accuracy or integrity of any part of the work are appropriately investigated and resolved.

## Conflict of Interest Statement

The authors declare that the research was conducted in the absence of any commercial or financial relationships that could be construed as a potential conflict of interest.

## Abbreviations

CPGs: central patterns generators
DL: dominant limb
DL-KT: dominant limb—Kinesio^®^ Tex
DL-NKT: dominant limb—without Kinesio^®^ Tex
IOIs: inter-onset intervals
IWFEs: isochronous wrist’s flexion-extensions
KT: Kinesio^®^ Tex
NDL: non-dominant limb
NDL-KT: non-dominant limb—Kinesio^®^ Tex
NDL-NKT: non-dominant limb—without Kinesio^®^ Tex
NKT: without KT
SD: standard deviation
*ρ*(1): lag-one autocorrelation
w*ρ*(1): windowed lag-one autocorrelations.
